# Arrhythmic expression signatures of circadian clock‐associated transcription factors and chronic circadian disruption contribute to advanced prostate cancer growth

**DOI:** 10.1002/ijc.70149

**Published:** 2025-09-15

**Authors:** Ria Chopra, Haolong Li, Wenjuan Xie, Daniel Hau Tak Lam, Franky Leung Chan

**Affiliations:** ^1^ School of Biomedical Sciences, Faculty of Medicine The Chinese University of Hong Kong Hong Kong China

**Keywords:** chronic circadian disruption, chrono‐chemotherapy, circadian clock‐associated genes, nuclear receptors, prostate cancer

## Abstract

Disruption of circadian rhythms due to night‐shift work is classified as a probable carcinogen for cancers of the breast, prostate, and colorectum by the International Agency for Research on Cancer. Global epidemiological studies link chronic circadian clock disruption to increased risk of prostate cancer via hormone and metabolic dysregulation. This study investigated and compared the circadian expression patterns of core‐circadian controlled genes (CCCGs) and nuclear receptors (NRs) under a normal 12‐h light/dark cycle in normal mouse prostate and advanced androgen‐insensitive prostate tumors derived from a transgenic mouse model of prostate adenocarcinoma (TGMAP). Our results showed that a total of eight CCCGs and 22 NRs exhibited rhythmic oscillations in the normal mouse prostate. In contrast, the rhythmic expressions of CCCGs and NRs were significantly disrupted in TGMAP prostate tumors, with a concurrent loss of androgen receptor expression. Circadian administration of cisplatin at a specific morning time point (chrono‐chemotherapy), as applied in TGMAP tumor‐bearing mice, demonstrated optimal antitumor efficacy, which correlated with the circadian rhythmic expression of DNA damage repair genes. Finally, we showed that chronic jet‐lag conditions could promote the oncogenic growth of hormone‐sensitive VCaP‐derived xenograft tumors, with a correlation to elevated serum androgen levels and increased expression of enzyme genes involved in intratumoral androgen biosynthesis. Together, this study demonstrated that advanced prostate tumors exhibited dysregulated circadian transcriptional networks, as shown by their disrupted expression of CCCGs and NRs. The potential therapeutic application of chrono‐chemotherapy in advanced prostate cancer management and the disruption of circadian rhythms under chronic jet‐lag conditions could enhance prostate cancer growth.

## Graphical abstract

What's new?

Circadian rhythm disruption potentially fuels prostate cancer progression. The mechanisms and signaling molecules contributing to hormonal tumorigenesis, however, remain largely undefined. In this study, circadian expression patterns of core‐circadian controlled genes (CCCGs) and nuclear receptors (NRs) were investigated in advanced androgen‐insensitive prostate tumors derived from a transgenic mouse model of prostate adenocarcinoma (TGMAP). Experiments reveal that the expression of CCCGs and many NRs oscillates rhythmically, with circadian patterns disrupted in TGMAP animals. In addition, conditions mimicking chronic jet‐lag promoted the growth of hormone‐sensitive VCaP cell‐derived xenograft tumors, demonstrating that chronic circadian disruption enhances aggressive prostate tumorigenesis via increased testosterone levels.

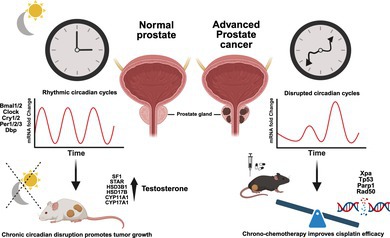

**Abbreviations**
ADT: Androgen‐deprivation therapy
AR: Androgen receptor
CCCGs: Core‐circadian controlled genes
CJL: Chronic jet‐lag
mCRPC: Metastatic castration‐resistant prostate cancer
NEPC: Neuroendocrine prostate cancer
NER: Nucleotide excision repair
NLD: Normal light–dark
NRs: Nuclear receptors
PCa: Prostate cancer
SCN: Suprachiasmatic nucleus
TGMAP: Transgenic mouse model of prostate adenocarcinoma
TTFL: Transcription‐translation‐feedback loop

## 1 INTRODUCTION

In mammals, the suprachiasmatic nucleus (SCN) in the hypothalamus acts as the central pacemaker in regulating the circadian clock. Its rhythmic neuronal and physiological control is influenced by and aligned with the daily light/dark cycle. With an almost 24‐h periodicity, this master circadian clock directs the cellular circadian rhythmic expressions of certain core‐circadian‐controlled genes (CCCGs) through a transcription‐translation‐feedback loop mechanism (TTFL), primarily to regulate the internal networks of systemic and cellular physiological and metabolic processes in peripheral tissues and organs.^1^ This circadian timekeeping mechanism enables peripheral organs to act as circadian oscillators, regulating and coordinating multiple physiological and metabolic functions, including endocrine, metabolic, and immune processes, to adapt and align with the 24‐h daily environmental changes in normal functions as well as in diseases.^2^ It is shown that disruption of circadian rhythms and daily metabolic activities due to sleep disorder, insomnia, and rhythm‐disrupting activities (e.g., night‐shift work) is closely associated with the development and progression of some cancers, particularly hormone‐dependent breast and prostate cancers.^3^ However, the perturbed mechanisms and signaling biomarkers in the disrupted metabolic and cellular rhythms contributing to hormonal tumorigenesis remain largely undefined. At the cellular level, mutual crosstalk between CCCGs and certain oncogenic pathway‐related genes can contribute to the progression of hormone‐dependent cancers.^3^ However, there is still a significant knowledge gap in understanding the potential roles of CCCGs and their perturbed rhythmic expressions towards the advanced development of prostate cancer, particularly the therapy‐resistant or hormone‐independent stage.

**FIGURE 1:**
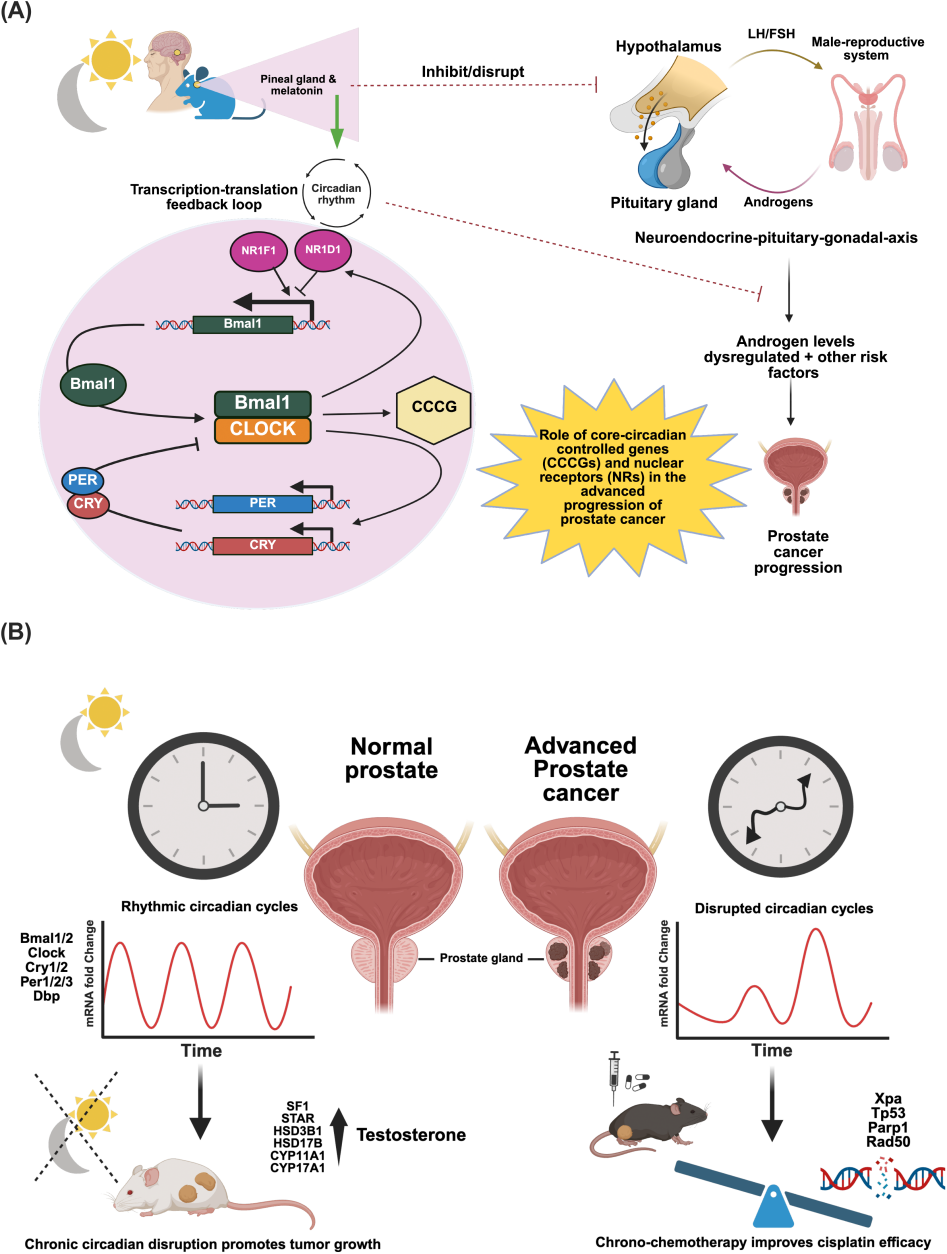
(A) Schematic diagram shows the systemic interactions between disrupted circadian clock regulation and the progression of hormone‐dependent prostate cancer. A bidirectional transcriptional regulatory loop exists between the circadian rhythms and the hypothalamic–pituitary‐gonadal axis, controlling androgen‐dependent prostate cancer. The transcription‐translation feedback loop (TTFL) in the regulation of circadian rhythms comprises the CCCGs, which are potentially involved in the advanced progression of prostate cancer. (B) The schematic diagram depicts the significance of chronic circadian disruption in enhanced oncogenic growth of prostate cancer via upregulation of steroidogenic regulatory NR (SF‐1) and enzyme genes, and the potential therapeutic application of specific time point cisplatin in chrono‐chemotherapy for optimal treatment efficacy, with its mechanistic link to the circadian expression of DNA damage repair genes. Created in BioRender. Chopra, R. (2025) https://BioRender.com/beqwia2.

In the positive feedback loop of TTFL, the CLOCK‐BMAL1 heterodimer (a basic helix–loop–helix transcription factor) binds to the E‐box enhancer elements upstream of Period (PER1 and PER2) and Cryptochrome (CRY1 and CRY2) genes (Figure 1A). This binding initiates the heterodimerization of PER and CRY proteins, which then recruit casein kinase one epsilon (CSNK1E) to indirectly repress their transcription by interacting with the BMAL1‐CLOCK complex in a negative feedback loop. A secondary feedback loop of TTFL is also formed by two nuclear receptors, RORα/β/γ (NR1F1/2/3) and REV‐ERBα/β (NR1D1/2), which bind competitively to the ROR response element (RORE) in the BMAL1 promoter to initiate BMAL1 expression. Notably, dysregulation of certain CCCGs, including multiple genomic variants of CRY1/2, CSNK1E, and NPAS2, is shown to be associated with an increased risk of prostate cancer.^4^ Other key CCCGs, including BMAL1, CLOCK, PER1/2/3, and RORα, are also shown to be associated with an increased risk of prostate cancer.^5^ These correlation studies suggest that disrupted circadian rhythms or dysregulation of CCCGs is implicated in prostate cancer development. Nonetheless, the impact of disrupted or uncoordinated circadian rhythmic expressions of CCCGs in the advanced development of prostate cancer is still largely undefined.

Disruption of circadian rhythms due to night‐shift work is classified as a probable carcinogen (Group 2A) for cancers of the breast, prostate, and colorectal by the International Agency for Research on Cancer.^6^ In a Canada–Japan–Sweden population‐based cohort study, chronic circadian rhythm disruption through night‐shift work and chronic jet‐lag conditions is implicated as high‐risk factors in prostate cancer.^7^, ^8^ A longitudinal study on sleeping duration conducted in Japanese men shows that men who slept 6 h or less have an increased incidence rate of metastatic prostate cancer than men who slept at least 9 h or more.^9^ These correlations between disrupted circadian rhythms and prostate cancer development are attributed to lower melatonin release in smaller sleep duration, which in turn affects sex hormone secretion and exerts pro‐tumorigenic effects on prostate cancer cells.^10^ Circadian clock disruptions can also lead to alterations in androgen production and overall systemic testosterone levels, which can potentially impact the malignant growth of prostate cancer.^11^ On the other hand, androgens can regulate circadian responses in male mammals,^12^ creating a two‐way crosstalk between circadian clock disruption and androgen regulation. While the connection between circadian rhythmic disruption and the risk of prostate cancer is recognized, the specific metabolic activities and oncogenic pathways involved remain poorly understood.

Nuclear receptors (NRs) constitute a superfamily of transcription factors, and many members act as key metabolic sensors to control diverse metabolic activities, particularly carbohydrate and lipid metabolism in the peripheral metabolic tissues in a circadian manner.^13^ Previously, we have demonstrated that NRs, LRH‐1, and ERRα can support intratumoral androgen biosynthesis in advanced prostate cancer by transcriptionally regulating multiple key steroidogenic enzyme genes involved in androgen biosynthesis, thereby enhancing androgen signaling in prostate cancer.^14^, ^15^ Besides functioning as cellular regulators of CCCGs, Rorα and Rev‐erbα can regulate murine Ar gene transcription and testosterone levels, suggesting that crosstalk between these NRs could play roles in prostate cancer progression.^16^, ^17^

Chronic circadian disruption negatively impacts circulating testosterone. Previous studies suggest that certain CCCGs and NRs are implicated in prostate cancer development.^5^ Hence, this study aims to define the circadian rhythmic expression profiles of CCCGs and NRs in a transgenic mouse model of hormone‐independent prostate cancer (TGMAP) compared to normal mouse prostates. Secondly, to better understand the significance of a disrupted circadian clock, we investigated the impact of chronic jet‐lag conditions on prostate cancer growth using a hormone‐sensitive VCaP‐derived xenograft model of prostate cancer to provide insights into the complex interplay between circadian rhythmic disruption, circulating testosterone levels, and metabolic targets in the malignant growth of prostate cancer.

## 2 MATERIALS AND METHODS

### 2.1 Mouse prostates and TGMAP tumor allograft model

Male C57BL/6J mice aged 8 to 10 weeks, provided by the CUHK Laboratory Animal Services Centre, were used for the circadian rhythms‐associated expression profiling study. Mice were entrained to normal 12‐h light/dark cycles (7:00–19:00 with lights on and 19:00–7:00 with lights off) and provided with water and standard rodent chow ad libitum at a controlled temperature. Mice were CO_2_‐euthanized at 4‐h intervals over 24 h, with three mice per time point. Dorsal and ventral prostate tissues were dissected and quickly stored in TRIZOL at −80°C or frozen in liquid N_2_ until RNA extraction for real‐time quantitative qRT‐PCR analysis.

We previously established a transgenic mouse prostate adenocarcinoma model (TGMAP) based on the prostate‐specific induction of oncogenic SV40‐Tag expression by targeting the PSP94 gene.^18^ Using this model, one tumor allograft line was established from the prostate tumors developed in a 13‐month‐old mouse, and the tumor allografts could grow in either intact or castrated syngeneic C56BL/6 J mice. The tumor allografts were pathologically characterized as poorly differentiated, displaying small cell morphology and neuroendocrine phenotypes with suppressed androgen receptor (AR) signaling. TGMAP tumor fragments of 2mm^3^ sizes were implanted subcutaneously into the flanks of young adult C57BL/6J male mice (8–10 weeks) and allowed to grow for 9 to 10 weeks. Mice bearing implanted allograft tumors were entrained to normal 12‐h light/dark cycles as described above and provided with water and standard rodent chow ad libitum at a controlled temperature. After 9 to 10 weeks, mice bearing tumor allografts were sacrificed at 4‐h intervals over 24 h with at least three mice per time point. The tumor allografts were dissected and quickly stored in TRIZOL at 80°C.

### 2.2 Cisplatin chronotherapy treatment

Male C57BL/6J mice, aged 8 to 10 weeks, were used for the allograft growth of TGMAP tumors. TGMAP allograft tumors of size 2‐mm^3^ were transplanted in the subcutaneous flanks of mice for allograft growth. Mice were entrained to normal 12‐h light/dark cycles as described above and provided with water and standard rodent chow ad libitum at a controlled temperature. Cisplatin (MCE, Cat #HY‐17394) was freshly prepared in 1 × PBS solution. After 30 days, tumor‐bearing mice were divided into three groups for intraperitoneal injections of cisplatin (5 mg/kg) and PBS (control), administered at 8 am, 3 pm, or 8 pm, respectively. After a total of three doses, once every 4 days, the respective mice were then sacrificed at 8 am, 3 pm, or 8 pm, respectively. The allograft tumors were dissected and stored in TRIZOL at −80°C for RNA extraction.

### 2.3 Chronic jet‐lag circadian rhythms experiments

We used a previously established androgen‐sensitive human prostate cancer cell VCaP‐based xenograft model for the study of chronic circadian rhythmic disruption.^14^ The VCaP cell line was authenticated by short tandem‐repeat (STR) profiling and confirmed to be Mycoplasma‐free before use. In brief, VCaP cells (2 × 10^6^ cells/100 μL mixed 1:1 in Matrigel) were injected subcutaneously into the flanks of 6–8‐week‐old intact male NSG mice. Mice bearing the injected VCaP cells were randomly housed into two groups under either a normal 12‐h light/dark cycle or chronic jet‐lag conditions. For the chronic jet‐lag conditions of an 8‐h advance shift in the light cycle every 2 days,^19^ mice were housed in IVC cages with a battery‐controlled programmable LED system (TECHNIPLAST Digital Ventilated cages [DVC]® Leddy) at a room temperature of 25°C and humidity of 45–65% with food and water provided ad libitum. Once the tumor xenografts were palpable, electronic calipers were used to measure their growth using the formula (length x width^2^ x 0.5).

### 2.4 Reverse transcription and quantitative real‐time qRT‐PCR analysis

Total RNA was extracted from normal mouse prostates and tumor samples, pre‐treated with DNase I, and reverse transcribed to cDNA (PrimeScript™ RT reagent kit, Takara). Quantitative real‐time qRT‐PCR for the analysis of mRNA levels of target genes was performed in a real‐time PCR system (QuantStudio™ 7 Pro Real‐Time PCR System, Applied Biosystems) using a SYBR‐Green‐based comparative ΔΔCt method (TB Green Premix Ex Taq Tli RNase H Plus, Takara) and normalized against β‐actin. The specific validated PCR primers used for the studied genes are listed in Tables S1–S4.

### 2.5 Testosterone measurement

Before the mice were sacrificed, fresh blood samples were collected in 1.5 mL tubes and allowed to clot at room temperature for 30 min. The blood samples were centrifuged at 10^3^×*g* for 15 min at 4°C. The upper layer of serum was removed and immediately stored in a fresh 1.5 mL tube at −20°C for further use. Testosterone levels in blood samples from mice were measured by competitive enzyme immunoassay (Testosterone Parameter Assay Kit, #KGE010, Bio‐Techne R&D SYSTEMS) according to the manufacturer's instructions.

### 2.6 Statistical analysis

Student's *t*‐test and analysis of variance (ANOVA) were used to express mean ± SD for continuous variables. The differences were analyzed with a significance of *p* < 0.05.

## 3 RESULTS

### 3.1 Circadian rhythmic expression profiles of core‐circadian controlled genes and nuclear receptors in mouse prostates

**FIGURE 2:**
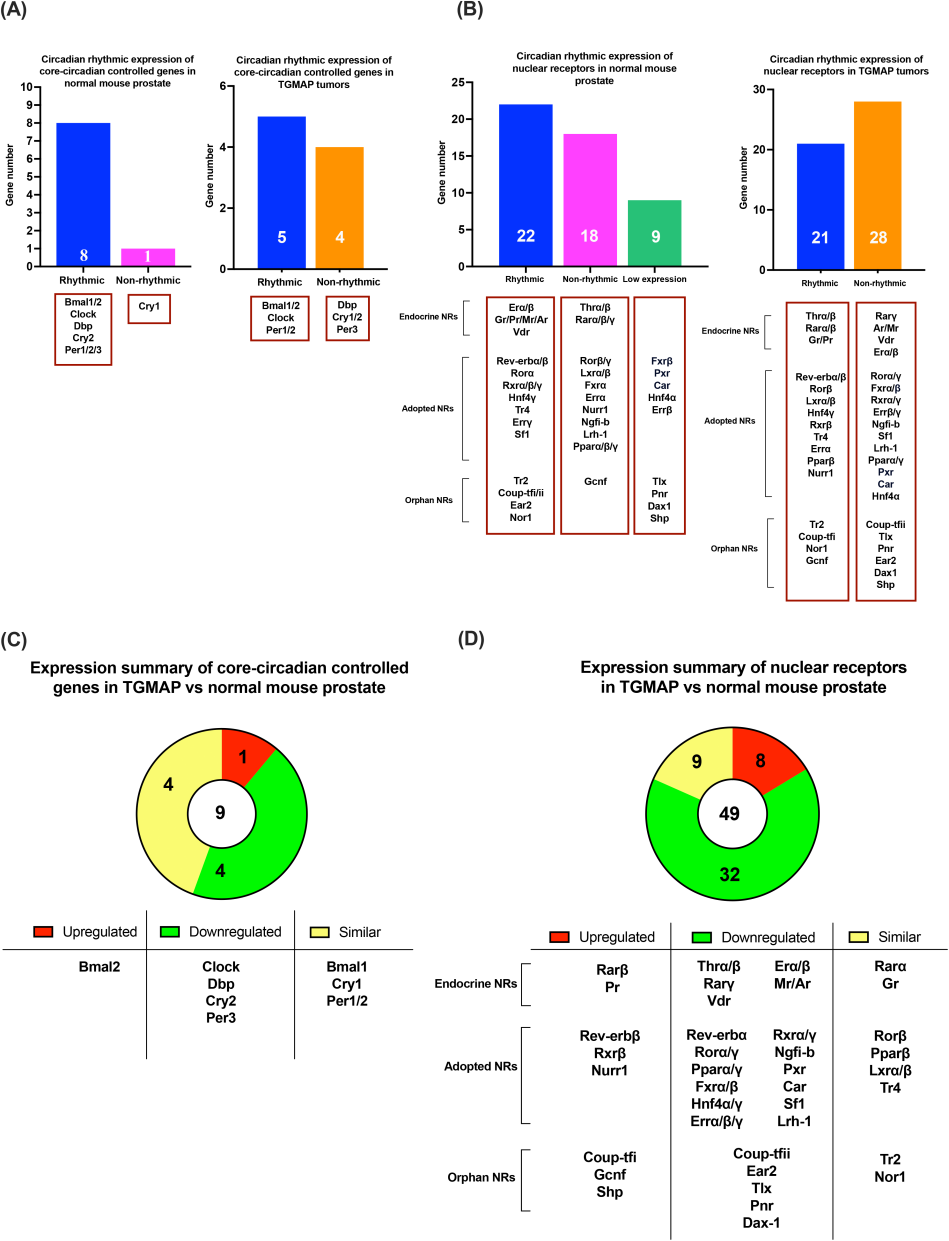
Circadian rhythmic profiles of core‐circadian controlled genes (CCCGs) and nuclear receptors (NRs) expressions in normal mouse prostates and TGMAP prostate tumors. (A and B) Bar charts summarize the distribution of expression profiles of CCCGs and NRs regarding their circadian rhythmicity, as observed in normal mouse prostates and TGMAP prostate tumors. The expressed NRs are grouped as endocrine, adopted, and orphan, according to their ligand dependence or independence. Among the detected CCCGs and NRs, 8 CCCGs and 22 NRs exhibited rhythmic expression patterns. Nine NRs showed either low or undetectable expressions in normal prostates. (C and D) Doughnut charts summarize the mRNA expression levels of CCCGs, and NRs detected in TGMAP tumors as compared to those in normal mouse prostates. Among the detectable NRs that show either rhythmic or non‐rhythmic expression patterns in normal prostates, 5 NRs (Fxrβ, Hnf4α, Errβ, Pxr, and Car) are undetectable in TGMAP tumors. Relative mRNA expression fold changes of CCCGs and NRs are normalized to endogenous β‐Actin. Results are presented as mean ± SD of three independent samples.

**FIGURE 3:**
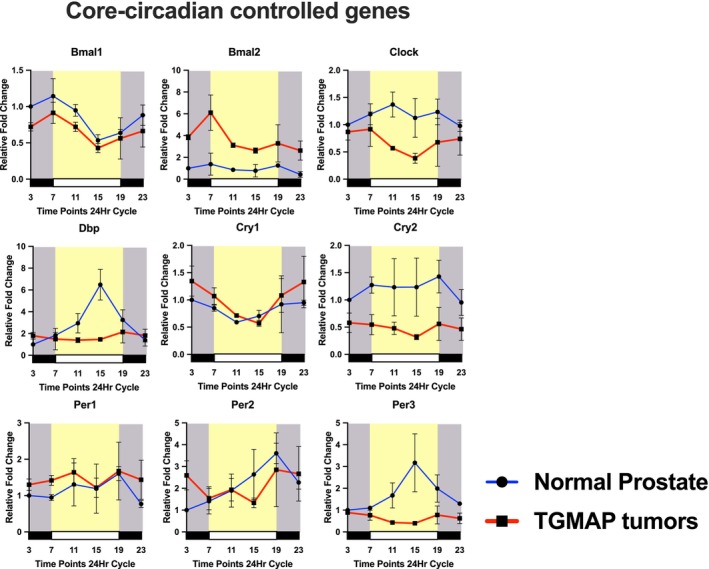
Circadian rhythmic expression profiles of CCCGs in normal mouse prostates (blue lines) versus their corresponding expressions in TGMAP prostate tumors (red lines) across the day cycles. Mice were entrained to 12‐h light (07–19) and 12‐h dark (19–07) cycles. Samples of normal prostates and TGAMP tumors (*n* = 3) were collected at 4‐h intervals throughout the 12‐h light/dark cycles for qRT‐PCR analysis. Relative mRNA expression fold changes of CCCGs were normalized to that of endogenous β‐Actin. Results are presented as the mean ± SD of three independent samples.

**FIGURE 4:**
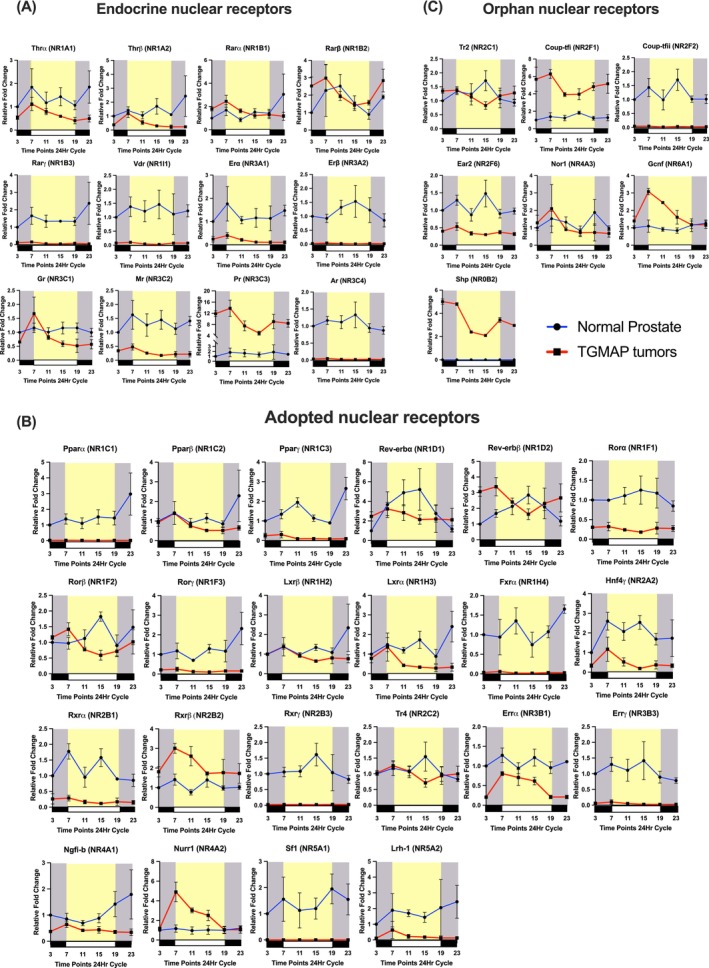
Circadian rhythmic expression profiles of NR expressions in normal mouse prostates (blue lines) and TGMAP prostate tumors (red lines). mRNA expression profiles of NRs are categorized according to their ligand dependence or independence as (A) endocrine NRs, (B) adopted NRs, and (C) orphan NRs. Relative mRNA expression fold changes of NRs are normalized to that of endogenous β‐Actin. Results are presented as the mean ± SD of three independent samples.

To provide insights into the significance of circadian rhythmic expressions of CCCGs and NRs in the functional maintenance and differentiation of normal mouse prostates, we compiled their individual expression profiles spanning over the normal 12‐h light/dark cycles in young adult C57BL/6J mice. Our RT‐qPCR analysis demonstrated that all nine major CCCGs and 40 members of NRs showed positive and detectable expressions in normal mouse prostates (Figure 2A–D), suggesting their diverse and broad regulatory roles in normal mouse prostates. To explore their functional implications in normal mouse prostates and link expressions to peripheral circadian oscillations, we further analyzed their circadian rhythmic expression patterns throughout the normal 12‐h light/dark cycles (Figures 3 and 4A–C). Among the nine CCCGs, eight CCCGs exhibited distinct circadian rhythmic expression patterns, including Bmal1, Bmal2, Clock, Dbp, Cry2, and Per1/2/3 (Figures 2A and 3). Among the 40 expressed NRs, 22 NRs (representing about 55% of the expressed NR superfamily) displayed circadian rhythmic expression patterns, while 18 NRs showed non‐rhythmicity in normal mouse prostates (Figures 2B and 4A–C). About nine NRs showed either low or undetectable expression levels in normal mouse prostates. The 22 NRs displaying circadian rhythmic expressions can be categorized into three subgroups according to their ligand‐dependence or ‐independence: (a) 7 hormone or endocrine NRs, including Erα, Erβ, Gr, Pr, Mr, Ar, and Vdr; (b) 10 adopted NRs, including Rev‐erbα, Rev‐erbβ, Rorα, Rxrα, Rxrβ, Rxrγ, Hnf4γ, Tr4, Errγ, and Sf1; and (c) 5 orphan NRs, including Tr2, Coup‐tfi, Coup‐tfii, Ear2, and Nor1 (Figure 2B). Among these NRs, Rev‐erbα/β and Rorα/β/γ are characterized to function as CCCGs.^20^ We further categorized the circadian rhythmic expression profiles of NRs in the normal prostates into three rhythmic patterns based on their mRNA expression peaks: (a) 1‐peak pattern (including Erα, Ar, Rev‐erbα, Rev‐erbβ, Rorα, Rxrγ, Tr4), (b) 2‐peaks pattern (Rarβ, Rarγ, Erα, Gr, Pr, Pparβ, Pparγ, Rorβ, Fxrα, Hnf4γ, Rxrα, Rxrβ, Errα, Sf1, Tr2, Coup‐tfI, CouptfII, Ear2, Nor1), and multiple peaks pattern (Thrα, Thrβ, Rarα, Rarβ, Vdr, Mr, Pparα, Rorγ, Lxrβ, Lxrα). The mRNA expression peaks implicate the relative potential activities of the genes expressed in the mouse prostates at a specific time point. The circadian rhythmic expression patterns, as displayed by multiple CCCGs and NRs, reflect their diverse roles in the transcriptional regulation of the circadian clock and endocrine and metabolic control in the mouse prostate gland.

### 3.2 Circadian rhythmic expressions of core‐circadian controlled genes and nuclear receptors in TGMAP mouse prostate tumors

To explore whether the CCCGs and NRs would display altered and disrupted expression patterns regarding their circadian rhythmicity and expression levels in prostate tumors, we next examined their circadian expression profiles in TGMAP prostate tumors developed in host mice housed in the same normal 12‐h light/dark cycles. A total of nine CCCGs and 40 NRs showed detectable expressions in TGMAP tumors. Analysis of their circadian expression patterns revealed that five CCCGs (Bmal1 and Bmal2, Clock, Per1, and Per2) and 21 NRs (including 6 endocrine NRs, 11 adopted NRs, and 4 orphan NRs) (Figure 2A,B) displayed rhythmic expression patterns. Analysis of the relative expression levels of CCCGs showed that one CCCG (Bmal2) exhibited consistent up‐regulation, four CCCGs (Clock, Dbp, Cry2, and Per3) with down‐regulation patterns, and four CCCGs (BmaI1, Cry1, Per1, and Per2, Rorβ) with similar expression levels as compared to normal mouse prostates (Figure 2C). Among the down‐regulated CCCGs, Dbp and Per3 lost their circadian rhythmic expression patterns, and Clock showed a disrupted pattern in TGMAP tumors compared to normal prostates (Figure 3).

Similar analyses of NR expression levels performed in TGAMP tumors demonstrated that eight NRs exhibited upregulation, 32 NRs showed downregulation (including a significant loss of Ar), and nine NRs had similar expression levels compared to normal mouse prostates (Figure 2D). Further analysis of those NRs with upregulation patterns in TGAMP tumors revealed that three NRs (Rarβ, Nurr1, and Gcnf) exhibited rhythmic expression patterns in TGAMP tumors but not in normal prostates, and four NRs (Pr, Rev‐erbβ, Rxrβ, and Coup‐tfi) showed similar rhythmic expression patterns in both TGAMP tumors and normal prostates (Figures 2D and 4A–C). Among the detectable NRs that showed either rhythmic or non‐rhythmic expression patterns in normal prostates, five NRs (Fxrβ, Hnf4α, Errβ, Pxr, and Car) lost their expression in TGAMP tumors. Further analysis of the detected NRs expressed at different levels in TGAMP tumors revealed that these NRs with altered expression levels displayed disrupted rhythmic expression patterns compared to normal mouse prostates (Figure 4A–C). Interestingly, among these upregulated NRs, Shp, which showed undetectable expression in normal prostates, exhibited not only upregulation but also rhythmic expression patterns in TGAMP tumors (Figure 4C).

### 3.3 Circadian administration of cisplatin at different daily time points could exert improved antitumor efficacy on TGMAP prostate tumors

**FIGURE 5:**
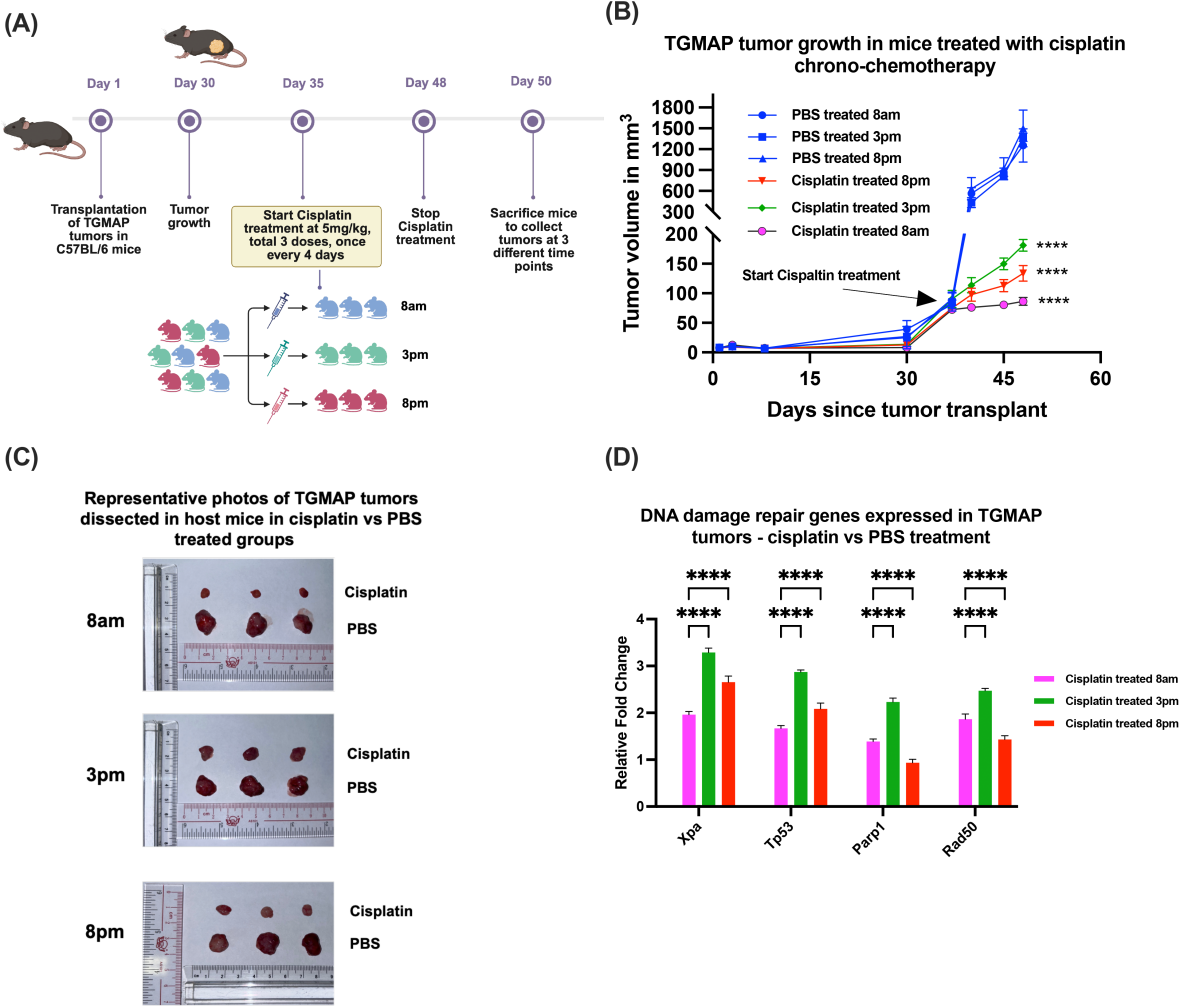
Circadian administration of cisplatin at different daily time points can induce improved antitumor impact on TGMAP prostate tumors. (A) Schematic diagram illustrates the protocol of circadian delivery of cisplatin at different time points (8 am, 3 pm, and 8 pm) on randomly selected host C57BL/6J mice bearing the TGMAP tumors (*n* = 3 per group). Created in BioRender. Chopra, R. (2025) https://BioRender.com/vmeqepw. (B) The tumor growth curve shows the growth of TGMAP tumors (volume in mm^3^) grown upon cisplatin or PBS treatment, administered at different daily time points throughout the treatment period. Results showed that cisplatin‐treated TGMAP tumors grew significantly smaller than those treated with PBS, with the most pronounced antitumor effect observed when cisplatin was administered at 8 am compared to 3 pm and 8 pm. (****p < 0.0001 cisplatin vs. PBS, two‐way ANOVA). (C) Images show the dissected TGMAP tumors harvested at the end of the experiment (Day 50) after cisplatin or PBS treatment, delivered at different daily time points. (D) Relative mRNA expression levels of 4 key DNA damage repair genes (Xpa, Tp53, Parp1, and Rad50) expressed in TGMAP tumors upon cisplatin treatment, as compared to PBS treatment being delivered at different time points. Results showed that all four DNA damage repair genes expressed their highest levels in TGMAP tumors when cisplatin was administered at 3 pm, compared to other time points. (****p < 0.0001 cisplatin vs. PBS, two‐way ANOVA).

Platinum‐based chemotherapy is the mainstay treatment option for neuroendocrine prostate cancer (NEPC), which is characterized by its resistance to AR‐targeted therapy due to the loss of AR signaling and positive expression of neuroendocrine biomarkers.^21^ It is demonstrated that the cellular DNA repair system, in response to cisplatin‐induced DNA damage, is under tight circadian control in murine tissues and organs.^22^ Based on the functional connection between circadian clock genes and the cell cycle, as well as oncogenes, chrono‐chemotherapy, or the circadian administration of drugs at specific daily time points, is suggested for cancer treatment, as it may offer maximum efficacy and minimal side effects.^23^, ^24^ Hence, we sought to evaluate whether circadian administration of cisplatin could offer improved efficacy for the chemotherapy of androgen‐insensitive prostate cancer using the NEPC‐like TGMAP allograft tumor model (Figure 5A). Results of in vivo tumor growth showed that cisplatin administration at 8 am offered the best antitumor efficacy on TGMAP allograft tumors compared to administration at 8 pm and 3 pm, respectively (Figure 5B,C). To provide further insights into the potential involvement of the circadian clock in the DNA repair program, we also analyzed the circadian mRNA expression patterns of four key DNA damage repair genes (Xpa, Tp53, Parp1, and Rad50) in TGMAP tumors upon time‐specific administration of cisplatin. Expression analysis revealed that all four DNA damage repair genes exhibited their highest expression peaks in TGMAP tumors upon cisplatin administration at 3 pm, which implies that cisplatin administration at 3 pm shows a close association with the least cisplatin‐induced antitumor efficacy as compared to drug administration at 8 am and 3 pm, respectively (Figure 5D). Results also showed that both Xpa and Tp53 exhibited the lowest expression levels in TGMAP tumors upon cisplatin administration at 8 am, implicating that this specific administration time point could offer the optimal chemotherapy effect on TGMAP tumors.

### 3.4 Chronic jet‐lag condition could promote in vivo tumorigenicity of hormone‐sensitive VCaP‐derived tumor xenografts

**FIGURE 6:**
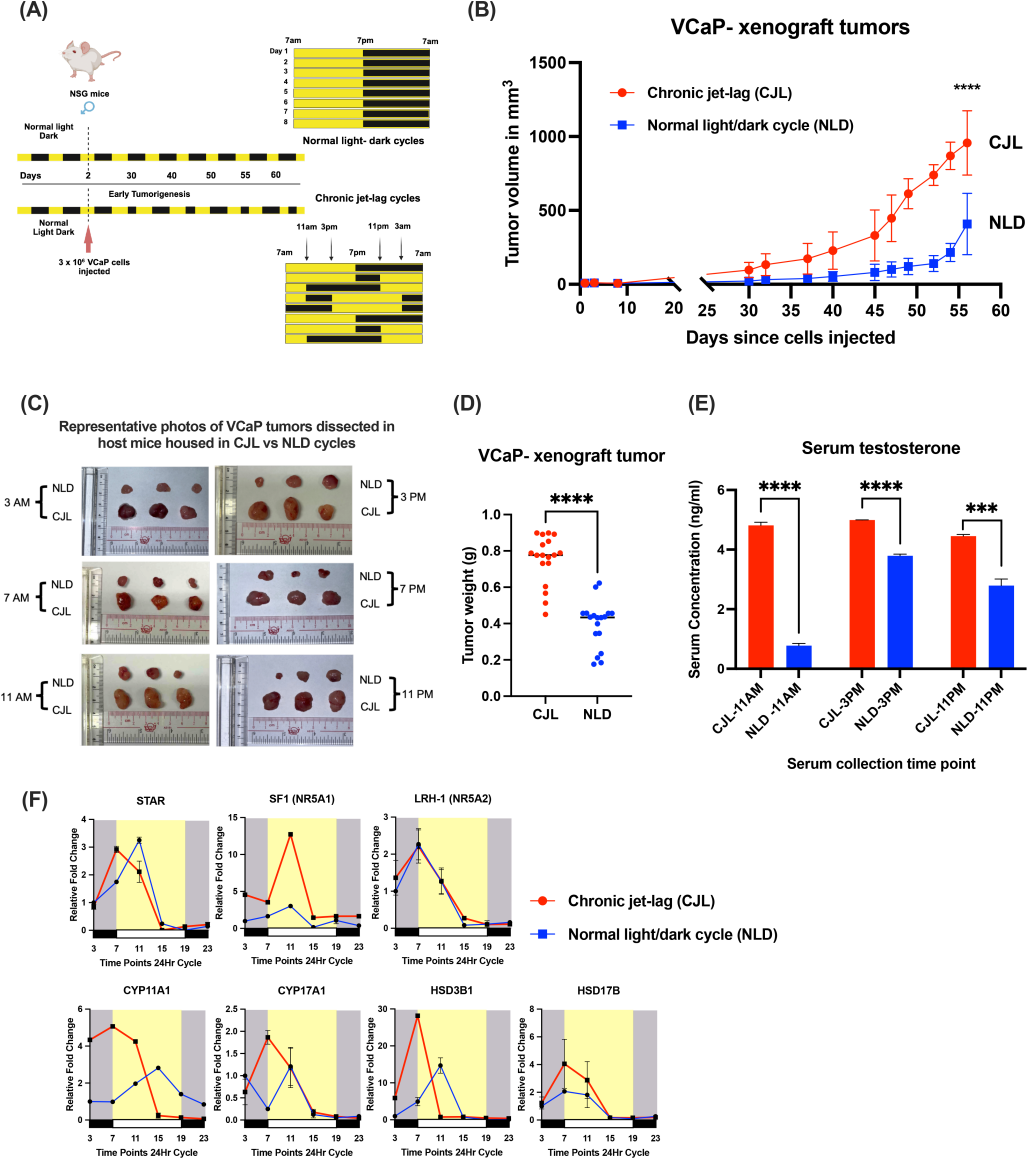
Chronic jet‐lag conditions can promote in vivo tumorigenicity of hormone‐sensitive VCaP‐derived prostate xenograft tumors. (A) Schematic diagram illustrates the experimental scheme of NSG host mice bearing VCaP xenograft tumors being housed under the chronic jet‐lag conditions (CJL) or normal 12‐h light/dark cycles (NLD) for 8 weeks. Created in BioRender. Chopra, R. (2025) https://BioRender.com/a5jsqh1 (B) Tumor growth curve shows the growth of VCaP xenograft tumors grown in mice being housed under CJL or NLD conditions. (C) Represented images of VCaP tumors dissected from host mice housed under CJL or NLD conditions, harvested at different time points at the end of the experiments. (D) Graph shows the tumor weights of VCaP tumors harvested from mice housed under CJL or NLD conditions at the end of the experiments. Results showed that VCaP tumors grew at significantly larger sizes in mice housed under CJL than in NLD conditions. (****p < 0.0001 CJL vs. NLD, unpaired Student's *t*‐test) (E) Graph shows the serum levels of testosterone measured in blood samples collected at three different time points from tumor‐bearing mice housed under CJL or NLD conditions. Results showed that testosterone serum levels collected from mice housed under CJL conditions were higher than those from mice housed under NLD conditions. (****p < 0.0001 and ***p < 0.001 CJL vs. NLD, unpaired Student's *t*‐test) (F) Graphs show the circadian mRNA expression levels of two key nuclear receptors (SF‐1/NR5A1, LRH‐1/NR5A2) and five steroidogenic enzyme genes (STAR, CYP11A1, CYP17A1, HSD3B1, and HSD17B) involved in intratumoral androgen biosynthesis as expressed in VCaP tumors harvested at different daily time points in host mice housed under CJL or NLD conditions. Results showed that five steroidogenic enzyme genes displayed higher expression levels and commonly reached their peaks at 7 am in VCaP tumors grown in mice under CJL conditions than in mice under NLD conditions.

Epidemiological studies indicate that rotating shift work and sleep disorders are highly associated with the increased risk of prostate cancer.^7^, ^8^, ^9^ In this regard, we experimentally evaluated whether chronic jet‐lag conditions (CJL), mimicking disrupted circadian rhythms, could affect the in vivo tumorigenicity of prostate cancer using an established hormone‐sensitive human prostate cancer cell line, VCaP‐derived tumor xenograft model^14^ (Figure 6A). Results showed that VCaP‐derived tumor xenografts grew significantly larger in host mice housed under the CJL conditions than in the normal 12‐h light/dark cycle (NLD) conditions (Figure 6B–D). Analysis of serum testosterone levels by enzyme immunoassay revealed that host mice housed under CJL conditions consistently had higher circulating testosterone levels in blood collected at various time points throughout the day compared to mice housed in NLD conditions (Figure 6E). To provide further insights into whether VCaP xenograft tumors grown under CJL conditions exhibit dysregulated intratumoral androgen biosynthesis, we examined the mRNA expression of key regulators and steroidogenic enzyme genes involved in this process. Results showed that a key nuclear receptor, SF1 (NR5A1), involved in steroidogenesis^25^ displayed significantly higher expression levels, reaching an expression peak at 11 am in VCaP tumors grown under CJL conditions compared to tumors grown under NLD conditions (Figure 6F). Results also showed that another NR5A subfamily member, LRH‐1 (NR5A2), which plays a key role in the intratumoral androgen biosynthesis in CRPC,^14^ exhibited the same expression pattern in VCaP tumors grown under both CJL and NLD conditions. Furthermore, expression analysis also showed that the expressions of STAR (steroidogenic acute regulatory protein) and four key steroidogenic enzyme genes (HSD3B1, HSD17B, CYP11A1, and CYP17A1) expressed higher mRNA levels and peaks at 7 am in VCaP tumors grown under CJL conditions, in contrast to their corresponding expressions in tumors grown under NLD conditions (Figure 6F). These results suggest that long‐term disruption of circadian rhythms can lead to significant disturbance or elevation of systemic androgen levels and signaling, which could exert a considerable impact on the promotion of the oncogenic growth of prostate cancer.

## 4 DISCUSSION

This study highlights the circadian expression profiles of CCCGs and NRs in advanced prostate tumors derived from a transgenic mouse model of prostate adenocarcinoma (TGMAP) compared to normal mouse prostates. Most CCCGs and NRs exhibited downregulated rhythmic expression patterns in the TGMAP tumors, reflecting the loss of circadian homeostasis and dysregulation in transcriptional controls and metabolic balance in advanced prostate cancer. Notably, chrono‐chemotherapy treatment of TGMAP tumors shows that specific morning treatment with cisplatin limits tumor growth in vivo more efficiently than treatment either in the afternoon or evening. Furthermore, we demonstrate that chronic circadian clock disruption significantly impacts advanced hormone‐dependent prostate cancer progression, as evidenced by elevated serum levels of testosterone and increased expression of genes involved in intratumoral androgen biosynthesis (Figure 1B).

Studies on the temporal expression profiles of transcription factors CCCGs and NRs, as well as their transcriptional networks in cancers, including prostate cancer, are currently lacking. A survey study on circadian expression profiling of the NR superfamily has been conducted in several mouse active metabolic tissues (brown and white adipose tissues, liver, and skeletal muscle), suggesting their close links to metabolism.^13^ Our results showed that most of the CCCGs and NRs were positively expressed and displayed circadian rhythmic cycles in the normal mouse prostates (Figure 2A–D), suggesting that a stable circadian clock plays a significant role in maintaining metabolic oscillations and the normal functioning of the prostate gland. Among NRs, we detected that the major metabolic homeostasis regulators, peroxisome proliferator‐activated receptors (Pparα/β/γ), were positively expressed in the normal mouse prostates (Figure 4B), potentially contributing to the normal growth and differentiation of the prostate.^26^ Over the past two decades, there have been several attempts to decipher the roles of specific NRs in various cancers, including breast, liver, and colorectal cancer,^27^ while aberrations in selected clock genes have been studied in prostate cancer. Importantly, two key circadian‐regulating transcription factors, Bmal2 and Rev‐erbβ (Figures 2A–D and 3), exhibited upregulated expression patterns in TGMAP tumors throughout the 12‐h light/dark cycles, suggesting their potential roles in the growth of advanced prostate cancer. BMAL2 has been implicated as an oncogenic driver in multiple human cancers, including lung, colorectal, and breast cancer,^28^ where it can drive tumorigenesis and create an immunosuppressive micro‐environment for advanced tumor growth. Interestingly, a study using metastatic castration‐resistant prostate cancer (mCRPC) organoids derived from patients receiving androgen‐targeting therapies finds that while some AR target genes are significantly down‐regulated, the circadian clock gene BMAL2 is significantly upregulated,^29^ suggesting that its upregulation is linked to the loss of circadian rhythms in the advanced progression of prostate cancer. Furthermore, the CRY1 gene has been identified as a pro‐tumorigenic and hormone‐induced factor in mCRPC patients, with a characterized role in DNA repair in prostate cancer tumors.^30^ Simultaneously, REV‐ERBβ has been implicated as a transcriptional driver in treatment‐resistant prostate cancer through lineage plasticity towards NEPC phenotypes.^31^ In all, targeting the disrupted circadian regulators could offer potential therapeutic applications in advanced prostate cancer.

Our study showed that numerous NRs exhibited either loss of expression or circadian rhythmicity in NEPC‐like TGMAP tumors compared to normal mouse prostates, especially the loss of AR signaling, which may drive neuroendocrine trans‐differentiation and lineage plasticity in NEPC.^32^ However, multiple endocrine NRs (Rarβ and Pr), adopted NRs (Rxrβ and Nurr1), and orphan NRs (Coup‐tfi, Gcnf, and Shp) (Figure 2D), displayed differential and upregulated temporal expression patterns in the TGMAP tumors. AR, PR, and GR share significant homology in their DNA‐binding domains, suggesting that they could regulate common targets.^33^ In CRPC patients with developed resistance to ADT, tumor tissues are characterized by suppressed AR but increased GR expression^34^, implying that GR could substitute for and activate AR signaling in CRPC. Studies also show that while GR expression is reduced in primary prostate cancer, its expression becomes restored in mCRPC patients who are resistant to ADT.^35^ Similarly, PR expression is significantly associated with prostate cancer progression, and multivariate analysis verifies that PR may negatively drive patients to clinical failure.^36^ Given that these endocrine receptors share around 50% sequence homology in the ligand‐binding domain^37^ and PR shares an overall 87% sequence homology with the AR gene,^38^ they may activate AR‐regulated genes or replace AR in advanced progression to CRPC and NEPC. Our study revealed that TGMAP tumors exhibited significant upregulation of Rarβ, which is known to play a vital role in neural development and regeneration.^39^ However, it remains to be confirmed if RARβ may hold novel functions in neuroendocrine cancers, including NEPC. Among the orphan NRs, Coup‐tf1, Gcnf, and Shp exhibited significantly disrupted and upregulated expression patterns in TGMAP tumors compared to normal mouse prostate (Figure 4C). Human clinical models of prostate cancer validate that AR negatively regulates COUP‐TF1 at both the mRNA and protein levels.^40^ Similarly, expression and correlation analysis reveal that the orphan receptor GCNF (NR6A1) exhibits a notable positive association with both hormone‐sensitive clinical prostate cancer and CRPC.^41^ Meanwhile, orphan receptor SHP is characterized as a transcriptional repressor of AR.^42^ However, its role in NEPC remains to be determined.

Our study also highlights the potential application of chrono‐chemotherapy, linking the specific time‐point administration of cisplatin with the circadian rhythmic expression patterns of DNA damage repair genes to achieve optimal efficacy in the therapeutic management of hormone‐insensitive PCa. Pharmacokinetics studies have demonstrated a clear link between drug metabolism and circadian rhythms,^43^ indicating that timing androgen‐deprivation therapy (ADT) administration can improve efficacy and reduce toxicity in prostate cancer patients. ADT drugs can regulate lipid metabolism processes, which in themselves can rewire the prostate tumor microenvironment to desensitize it to endocrine targeting therapies.^44^ Since metabolic processes are regulated rhythmically at the cellular level,^13^ it is unclear what significant roles circadian clock genes play in prostate cancer's resistance to ADT. ADT can be prescribed to patients in a chronotherapeutic manner for improved outcomes, but drugging the intrinsic circadian clock can be a new gateway to overcome the drug resistance that is almost inevitable in these patients. Meanwhile, an aggressive subset of prostate cancer patients will become resistant to ADT therapies and develop therapy‐induced t‐NEPC,^32^ with unique features of AR loss and expression of neuroendocrine lineage markers. These NEPC patients are reliant on chemotherapy with carboplatin drugs.^45^ Studies in the last decade have identified that the nucleotide excision repair (NER) pathway is under tight circadian control, where this pathway involves several key components, including xeroderma pigmentosum (XPA),^46^ which functions as a CCCG, while DNA damage checkpoints and apoptosis are also under circadian control. The TP53 gene can induce an apoptotic response considering DNA damage,^47^ and it further modulates CCCGs such as PER2, which acts as a downstream effector in controlling TP53 function in cell cycle control and biological rhythms. Here, we identified that genes involved in the DNA‐damage repair mechanism upon cisplatin treatment were downregulated in the morning cisplatin‐treated TGMAP tumors compared to afternoon and evening treatments, while Parp1 and Rad50 showed the most downregulation in the night treatment group. The reduced NER and DNA damage repair capacity suggests that targeting tumor growth by cisplatin administration in the morning could offer optimal efficacy to hormone‐insensitive NEPC‐like TGMAP tumors. After treatment with platinum drugs such as cisplatin and even radiotherapy, the XPA gene is the rate‐limiting factor in the NER system that functions to eliminate bulky DNA lesions.^47^ Notably, NER is the only DNA repair mechanism involved, suggesting that other DNA repair pathways involved in mismatch repair, direct repair, base excision, and recombinant repair may not be involved in this experimental TGMAP model. The circadian chrono‐chemotherapy approach can offer more robust control of tumor growth since the NER‐related mechanisms are downregulated, and DNA damage caused by cisplatin cannot be repaired. However, further experiments are necessary to validate whether the rhythmicity of the XPA gene could function as a biomarker to personalize cisplatin treatment in clinical settings.

It is established that disruption of the central circadian clock control in the hypothalamic SCN can significantly impact the molecular coordination of rhythmic cellular processes in many key peripheral organs.^48^ However, the significance of circadian rhythm disruption on the growth of prostate cancer is still largely unknown. It is characterized that testosterone secretion and its subsequent activation of AR‐signaling are under strict rhythmic control,^49^ reflected by its diurnal fluctuation in circulating testosterone levels. Epidemiological studies show that circadian disruption due to night‐shift work and jet lag conditions can significantly increase the risk of prostate cancer development.^7^, ^8^, ^9^ Here, we established an experimental protocol that mimics chronic jet‐lag conditions to demonstrate the interconnection between the circadian clock and prostate cancer, via master circadian clock disruption in the SCN and the neuroendocrine‐pituitary‐gonadal axis.^19^ Circadian disruption could significantly promote the oncogenic growth of the androgen‐sensitive VCaP‐derived xenograft tumors, at least via elevated serum testosterone levels and enhanced expression of steroidogenic enzyme genes (Figure 6E,F). The elevated circulating testosterone levels observed in CJL‐treated mice further confirm peripheral circadian disruption, as compared to the rhythmic testosterone levels in NLD‐treated mice (Figure 6E). Our results showed that the key steroidogenic regulator STAR^14^ exhibited significantly altered mRNA expression, while nuclear receptor SF1(NR5A1) showed significant upregulation in CJL‐treated mice compared to that in NLD‐treated mice. In addition, several key enzyme genes involved in androgen biosynthesis, including HSD3B1, HSD17B, CYP11A1, and CYP17A1, showed marked expression disruption in tumors. Their peak expressions were exhibited in the morning hours in CJL‐treated mice, with patterns that contrasted sharply with tumors grown in NLD‐treated mice. Our findings are consistent with previous reports that CJL conditions can severely disrupt metabolic pathways,^1^, ^50^ leading to metabolic pathologies including obesity, diabetes, and hepatocellular carcinoma.

Since the present study was conducted in nocturnal mice, further validation is needed to determine the circadian rhythmic expression patterns of key transcription factors in the human prostate gland and prostate cancer. However, most circadian studies are confined to animal models, and collecting human clinical samples at different daily time points remains challenging. Uncoupling of circadian rhythmicity of transcription factors and NRs, as shown in TGMAP tumors, suggests that the loss of circadian homeostasis could contribute to the advanced growth of prostate cancer. Our study highlights the significance of disrupted circadian clock rhythms in the advanced growth of prostate cancer, at least in part through increased circulating androgen levels and dysregulated androgen biosynthesis. Moreover, our preclinical study also highlights the significance of circadian rhythm oscillations in chrono‐chemotherapy and their potential value for the better management of advanced NEPC patients undergoing chemotherapy. Ultimately, a deeper understanding of the circadian expression patterns of the CCCGs and NRs could facilitate the application of chronotherapy for more effective management of advanced prostate cancer.

## AUTHOR CONTRIBUTIONS

**Ria Chopra:** Conceptualization; investigation; writing – original draft; methodology; validation; formal analysis. **Haolong Li:** Methodology; formal analysis; resources. **Wenjuan Xie:** Methodology; investigation. **Daniel Hau Tak Lam:** Methodology; investigation. **Franky Leung Chan:** Conceptualization; investigation; writing – review and editing; methodology; formal analysis; resources; project administration; supervision.

## FUNDING INFORMATION

This study was supported direct Grant for Research (grant number: 2021.062), Chinese University of Hong Kong; General Research Fund (grant numbers: 1410322 and 14107623), National Natural Science Foundation of China and Research Grants Council Joint Research Scheme (grant number: N_CUHK402/20), Research Grants Council of Hong Kong.

## CONFLICT OF INTEREST STATEMENT

All authors declare no conflict of interest.

## ETHICS STATEMENT

All animal procedures were performed according to the institutional laboratory animal guidelines and with the approval of protocol number 22/174/DRG‐5‐C from the CUHK Animal Experimentation Ethics Committee.

## Supporting information


**DATA S1.** Supplementary tables.

## Data Availability

The data that support the findings of this study are available from the corresponding author upon reasonable request.
