# Supplementary material for: Arrhythmic expression signatures of circadian clock‐associated transcription factors and chronic circadian disruption contribute to advanced prostate cancer growth
Source: Int J Cancer. 2025 Sep 15;158(3):775–89. doi: 10.1002/ijc.70149 (PMC12670342; doi:10.1002/ijc.70149)
Supplement: Supplementary file 1 — DATA S1. Supplementary tables. [file IJC-158-775-s001.pdf]

Supplementary Materials

Arrhythmic expression signatures of circadian clock-associated transcription factors and chronic circadian disruption contribute to advanced prostate cancer growth

Ria Chopra, Haolong Li, Wenjuan Xie, Daniel Hau Tak Lam, Franky Leung Chan

Contents:

Supplementary Tables 1-4

Supplementary Table 1

Nuclear Receptor Name	Gene Name	Forward Primer (5'-3')	Reverse Primer (5'-3')
NR0B1	Dax1	AAGGGACCGTGCTCTTTAACC	TCTCCACTGAAGACCCTCAATGT
NR0B2	Shp	CGATCCTCTTCAACCCAGATG	AGGGCTCCAAGACTTCACACA
NR1A1	Thrα	GGATGGAATTGAAGTGAATGGAA	CCGTTCTTTCTTTTTCGCTTTC
NR1A2	Thrβ	CTCTTCTCACGGTTCTCCTC	AACCAGTGCCAGGAATGT
NR1B1	Rarα	CCAGCTTCCAGTCAGTGGTTA	TGCTCTGGGTCTCGATGGT
NR1B2	Rarβ	ACAGATCTCCGCAGCATCAG	GCATTGATCCAGGAATTTCCA
NR1B3	Rarγ	CCATGCTTTGTATGCAATGACA	TTCTGAATGCTGCGTCTGAAG
NR1C1	Pparα	ACAAGGCCTCAGGGTACCA	GCCGAAAGAAGCCCTTACAG
NR1C2	Pparβ	GCCTCGGGCTTCCACTAC	AGATCCGATCGCACTTCTCA
NR1C3	Pparγ	CAAGAATACCAAAGTGCGATCAA	GAGCTGGGTCTTTTCAGAATAATAAG
NR1D1	Rev-erbα	GGGCACAAGCAACATTACCA	CACGTCCCCACACACCTTAC
NR1D2	Rev-erbβ	TGGGACTTTTGAGGTTTTAATGG	GTGACAGTCCGTTCCTTTGC
NR1F1	Rorα	ACCGTGTCCATGGCAGAAC	TTTCCAGGTGGGATTTGGAT
NR1F2	Rorβ	GGCAGACCCACACCTACGA	CAGAGCCTCCCTGGACTTG
NR1F3	Rorγ	TCTACACGGCCCTGGTTCT	ATGTTCCACTCTCCTCTTCTCTTG
NR1H2	Lxrβ	AAGCAGGTGCCAGGGTTCT	TGCATTCTGTCTCGTGGTTGT
NR1H3	Lxrα	AGGAGTGTCGACTTCGCAAA	CTCTTCTTGCCGCTTCAGTTT
NR1H4	Fxr	TCCGGACATTCAACCATCAC	TCACTGCACATCCCAGATCTC
NR1I1	Vdr	GGCTTCCACTTCAACGCTATG	ATGCTCCGCCTGAAGAAAC
NR1I2	Pxr	CAAGGCCAATGGCTACCA	CGGGTGATCTCGCAGGTT
NR1I3	Car	GCTGCAAGGGCTTCTTCAG	AACGGACAGATGGGACCAA
NR2A1	Hnf4α	ACCAAGAGGTCCATGGTGTTT	GTGCCGAGGGACGATGTAG
NR2A2	Hnf4γ	ATGACCAGGTGGCCCTCTT	TGTAGCTCCAAGCAGCAGATG
NR2B1	Rxrα	CGGAACAGCGCTCACAGT	AGCTCCGTCTTGTCCATCTG
NR2B2	Rxrβ	CAAACGGCTCTGTGCAATCT	AGCCCTCGCAGCTGTAAAC
NR2B3	Rxrγ	GCCACCCTGGAGGCCTATA	AGCAGAAGCTTGGCAAACCT
NR2C1	Tr2	CGATCATGGCGACCATAGAA	ATGAACTGCTTGCCCTGTGT

Supplementary Table 1 (Continued)

Supplementary Table 1 (Continued)

NR2C2	Tr4	GTCATGAGTCTCTCCACCATCCT	GCTTTATCCGGTCACCAGAAA
NR2E1	Tlx	AGCCCGCCGGATCAA	CAAGCGTAGACCCCGTAGTG
NR2E3	Pnr	AGGTGATGCTAAGCCAGCATAG	GAGGAGCAATTTCCCAAACC
NR2F1	Coup-tfI	TGCTATTCACGTCAGATGCTTGT	CAGGGCACACTGTGATTTCTC
NR2F2	Coup-tfII	GCATGAGACGGGAAGCTGTAC	CGTTGGTCAGGGCAAACTG
NR2F6	Ear2	GAGGGCTGCAAGAGTTTCTTC	TCCGGTGGTGCTGATCAA
NR3A1	Erα	GCAGATAGGGAGCTGGTTCA	TGGAGATTCAAGTCCCCAAA
NR3A2	Erβ	GCCAACCTCCTGATGCTTCT	TCGTACACCGGGACCACAT
NR3B1	Errα	AGCAAGCCCCGATGGA	GAGAAGCCTGGGATGCTCTT
NR3B2	Errβ	CAGATCGGGAGCTTGTGTTC	TGGTCCCCAAGTGTCAGACT
NR3B3	Errγ	ACTTGGCTGACCGAGAGTTG	GCCAGGGACAGTGTGGAGAA
NR3C1	Gr	GCAAGTGGAAACCTGCTATGC	CATACATGCAGGGTAGAGTCATTCTT
NR3C2	Mr	AGCAGGCCTTTGAGGTCATT	AAGGCCCCACCATTCATG
NR3C3	Pr	GCTTGCATGATCTTGTGAAACA	TGTCCGGGATTGGATGAAT
NR3C4	Ar	TGTCAACTCCAGGATGCTCTACT	TGGCTGTACATCCGAGACTTG
NR4A1	Ngf1-b	ACGGTCCCTGCACAGCTT	ATGCGATTCTGCAGCTCTTC
NR4A2	Nurr1	GCACTTCGGCGGAGTTG	GGAATCCAGCCCGTCAGA
NR4A3	Nor1	AGTGTCGGGATGGTTAAGGAA	ACGACCTCTCCTCCCTTTCA
NR5A1	Sf1	CCCTTATCCGGCTGAGAATT	CCAGGTCCTCGTCGTACGA
NR5A2	Lrh-1	TGGGAAGGAAGGGACAATCTT	CGAGACTCAGGAGGTTGTTGAA
NR6A1	Gcnf	CCTCCCTCACAGTGTACAGCAA	TGTGATATAGGTAGATGAGTCGTTCAA

Supplementary Table 1

The table denotes the list of 5’-3’ forward and reverse primers used for RT-qPCR analysis of mRNA expression of 49 members of the murine nuclear receptor superfamily in the study

Supplementary Table 2

Gene	Forward (5'-3')	Reverse (5'-3')
β-actin	CATTGCTGACAGGATGCAGAAGG	TGCTGGAAGGTGGACAGTGAGG
Bmal1	CCAAGAAAGTATGGACACAGACAAA	GCATTCTTGATCCTTCCTTGGT
Bmal2	AAGGATGGTGCCTTCGTGACTC	GCCGCAATGAAGCAAAGACAGC
Cry1	CTGGCGTGGAAGTCATCGT	CTGTCCGCCATTGAGTTCTATG
Cry2	TGTCCCTTCCTGTGTGGAAGA	GCTCCCAGCTTGGCTTGA
Per1	TGGCTCAAGTGGCAATGAGTC	GGCTCGAGCTGACTGTTCACT
Per2	ATGCTCGCCATCCACAAGA	GCGGAATCGAATGGGAGAAT
Per3	GTGACAGCAGAGTCCCATGA	CACTGCCATCTCGAGTTCAA
Clock	TTGCTCCACGGGAATCCTT	GGAGGGAAAGTGCTCTGTTGTAG
Dbp	AATGACCTTTGAACCTGATCCCGCT	GCTCCAGTACTTCTCATCCTTCTGT

Supplementary Table 2

The table denotes the list of 5’-3’ forward and reverse primers used for RT-qPCR analysis of the mRNA expression of the murine core-circadian controlled genes in the study

Supplementary Table 3

Gene	Forward (5'-3')	Reverse (5'-3')
Xpa	GATCTGCCAACGTGTGACAG	GTGAATGGCGTGGGTTCTTCT
Tp53	ATGGAGTACGCATGGGGAC	GGCGAAGAACGAGAGAATGAAG
Parp1	CTCTCCCAGAACAAGGACGAAG	CCGCTTTCACTTCCTCCATCTTC
Rad50	TGGAGCAGGGTCGTCTACAG	CGCTCAAAACCATCCAATTCAAG

Supplementary Table 3

The table denotes the list of 5’-3’ forward and reverse primers used for RT-qPCR analysis of the mRNA expression of the murine DNA-damage repair genes in the study

Supplementary Table 4

Gene	Forward (5'-3')	Reverse (5'-3')
STAR	TACGTGGCTACTCAGCATCGAC	TCAACACCTGGCTTCAGAGGCA
SF1 (NR5A1)	CCAGACCTTCATCTCCATCGTG	TGGCGGTAGATGTGGTCGAACA
LRH-1 (NR5A2)	GGCTTATGTGCAAAATGGCAGATC	GCTCACTCCAGCAGTTCTGAAG
CYP11A1	TGGCATCCTCTACAGACTCCTG	CTTCAGGTTGCGTGCCATCTCA
CYP17A1	GCACACCAACTATCAGTGACCG	CCTTGTCCACAGCAAACTCACC
HSD3B1	GTCTTCGGTGTCACTCACAGAG	CTGGTGTAGATGAAGACTGGCAC
HSD17B	AGCCGATCTTCTCAGCACCAAG	CTGAAGCCACTGTGACGATGTG
β-actin	CATGTACGTTGCTATCCAGGC	CTCCTTAATGTCACGCACGAT

Supplementary Table 4

The table denotes the list of 5’-3’ forward and reverse primers used for the RT-qPCR analysis of the mRNA expression of human steroidogenic genes and enzymes
